# Assessing Protein Biomarkers to Detect Lethal Acute Traumatic Brain Injuries in Cerebrospinal Fluid

**DOI:** 10.3390/biom11111577

**Published:** 2021-10-25

**Authors:** Johann Zwirner, Simone Bohnert, Heike Franke, Jack Garland, Niels Hammer, Dustin Möbius, Rexson Tse, Benjamin Ondruschka

**Affiliations:** 1 Department of Anatomy, University of Otago, Dunedin 9016, New Zealand; 2 Institute of Legal Medicine, University Medical Center Hamburg-Eppendorf, 22529 Hamburg, Germany; dustin.moebius@uke.de; 3 Institute of Legal Medicine, University of Leipzig, 04103 Leipzig, Germany; 4 Institute of Forensic Medicine, University of Wuerzburg, 97078 Wuerzburg, Germany; simone.bohnert@uni-wuerzburg.de; 5 Rudolf Boehm Institute of Pharmacology and Toxicology, University of Leipzig, 04107 Leipzig, Germany; heike.franke@medizin.uni-leipzig.de; 6 Forensic and Analytical Science Service, NSW Health Pathology, Lidcombe 2141, Australia; jack.garland@health.nsw.gov.au; 7 Institute of Macroscopic and Clinical Anatomy, University of Graz, 8010 Graz, Austria; niels.hammer@medunigraz.at; 8 Department of Orthopedic and Trauma Surgery, University of Leipzig, 04103 Leipzig, Germany; 9 Fraunhofer IWU, 47720 Dresden, Germany; 10 Department of Forensic Pathology, LabPLUS, Auckland City Hospital, Auckland 1148, New Zealand; rexsont@adhb.govt.nz

**Keywords:** biomarker combination, glial fibrillary acidic protein, interleukin-6, post mortem biochemistry, traumatic brain injury

## Abstract

Diagnosing traumatic brain injury (TBI) from body fluids in cases where there are no obvious external signs of impact would be useful for emergency physicians and forensic pathologists alike. None of the previous attempts has so far succeeded in establishing a single biomarker to reliably detect TBI with regards to the sensitivity: specificity ratio in a post mortem setting. This study investigated a combination of body fluid biomarkers (obtained post mortem), which may be a step towards increasing the accuracy of biochemical TBI detection. In this study, serum and cerebrospinal fluid (CSF) samples from 30 acute lethal TBI cases and 70 controls without a TBI-related cause of death were evaluated for the following eight TBI-related biomarkers: brain-derived neurotrophic factor (BDNF), ferritin, glial fibrillary acidic protein (GFAP), interleukin 6 (IL-6), lactate dehydrogenase, neutrophil gelatinase-associated lipocalin (NGAL), neuron-specific enolase and S100 calcium-binding protein B. Correlations among the individual TBI biomarkers were assessed, and a specificity-accentuated threshold value analysis was conducted for all biomarkers. Based on these values, a decision tree modelling approach was performed to assess the most accurate biomarker combination to detect acute lethal TBIs. The results showed that 92.45% of acute lethal TBIs were able to be diagnosed using a combination of IL-6 and GFAP in CSF. The probability of detecting an acute lethal TBI was moderately increased by GFAP alone and considerably increased by the remaining biomarkers. BDNF and NGAL were almost perfectly correlated (*p* = 0.002; R^2^ = 0.944). This study provides evidence that acute lethal TBIs can be detected to a high degree of statistical accuracy using forensic biochemistry. The high inter-individual correlations of biomarkers may help to estimate the CSF concentration of an unknown biomarker, using extrapolation techniques.

## 1. Introduction

Traumatic brain injury (TBI) substantially contributes to the global injury burden with a continuously increasing prevalence over the last quarter of a century [1]. Approximately 11% of the 2.4 to 2.8 million annual TBI-related emergency department visits in the United States lead to hospitalization, with a lethal outcome in about a fifth of these cases [1,2]. Fluid biomarkers such as acute-phase proteins and specific proteins of the central nervous system (CNS) are an emerging objective tool to detect TBIs ante [3] and post mortem [4,5,6,7,8,9]. In a clinical setting, fluid biomarkers are promising, as (i) they may help detect TBIs in geriatric and pediatric patients, who account for a significant proportion of TBI-related emergency visits and may have limited ability to describe the underlying traumatic event [2]; (ii) they may prevent misdiagnosis of concussion, in which obvious external injury is often lacking and patient symptoms might not be characteristic [3]; and (iii) they may serve as a predictor for the outcome and mortality of a patient following TBI [10,11]. Apart from clinical applications, fluid biomarkers may help confirm TBIs in the field of forensic biochemistry [4,5,6,7,8,9,12,13]. Complementary to the post mortem confirmation of a TBI as a potential cause of death, fluid TBI-related biomarkers can be used for estimating time since death [8] and survival time [6,7,9] following the traumatic event.

However, despite numerous investigations of fluid TBI-related biomarkers, neither a single characteristic biomarker specific to TBI, nor a preferential body fluid has been established to date. Individual markers suffer either a poor sensitivity or a poor specificity, even though the respective counterpart often seems to be excellent [6]. This fact renders single fluid biomarkers unsuitable for use in TBI screening. Similar to clinical studies [14,15], a combination of TBI-related biomarkers might overcome this issue and increase the likelihood of accurately detecting TBIs. Previously, our group investigated brain-derived neurotrophic factor (BDNF) [8], glial fibrillary acidic protein (GFAP) [8], neuron-specific enolase (NSE) [6,9] and S100 calcium-binding protein B (S100B) [6,9] as comparatively specific CNS biomarkers in both TBI cases and controls. Similarly, the acute phase proteins ferritin [7], interleukin 6 (IL-6) [7], lactate dehydrogenase (LDH) [7] and neutrophil gelatinase-associated lipocalin (NGAL) [8] were investigated.

The overall aim of this study is to determine the combination of the aforementioned forensically investigated biomarkers (obtained post mortem) to detect lethal acute TBIs with the highest possible accuracy. This study aims to provide novel insights into the investigation of lethal TBIs for the emerging field of post mortem biochemistry [16,17,18,19]. This is the first study to provide a combined patient-matched evaluation of the aforementioned biomarkers, addressing the following hypothesis: a combination of fluid biomarkers sampled post mortem increases the accuracy of detecting an acute lethal TBI over any individual biomarker.

## 2. Material/Methods

### 2.1. Sample Collection, Assessment and Selection

**Figure 1:**
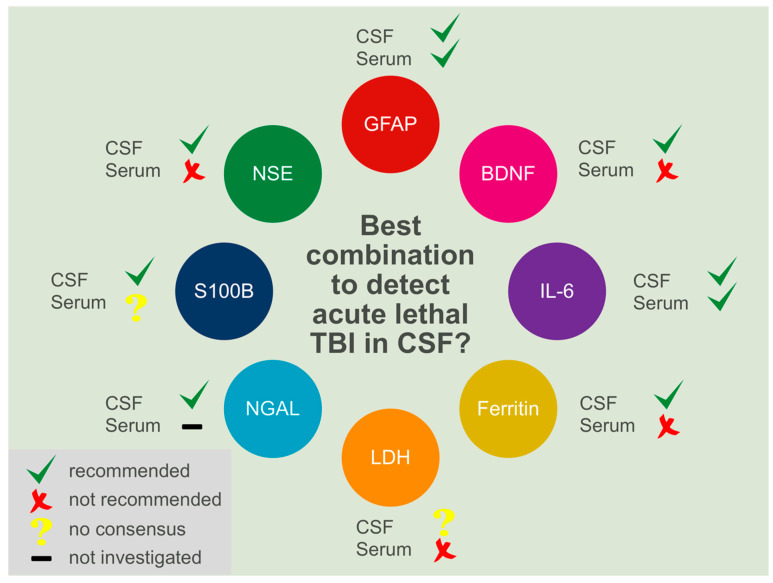
A graphical summary of previous post mortem studies by the authors [6,7,8,9] and other post mortem studies [4,5,12,13,20,21] for the biomarkers in this study, including a summary of whether these were considered to be useful for the detection of acute lethal TBIs. Most studies recommended the use of cerebrospinal fluid markers to investigate the respective TBI-related biomarkers compared to combined control groups. The use of serum markers was only recommended in a minority of studies.

Patient-specific serum and cerebrospinal fluid (CSF) sample data was extracted from the authors’ previous publications [6,7,8,9]. The inclusion criteria were an initial survival time of a maximum of 2 h following the traumatic event. Sample collection and processing were carried out as stated previously [6,7,8,9]. In brief, paired samples of serum and CSF were collected during routine forensic autopsies at the Institute of Legal Medicine of the University of Leipzig. Blood samples were taken from the femoral vessels and CSF samples from the suboccipital subarachnoid space. Both were taken using a sterile syringe, immediately centrifuged at 5000 rpm for 5 min at 4 °C and subsequently stored at −80 °C until further processing. The samples were allocated into two groups, a TBI group with 30 samples, and a non-TBI control group with 70 samples consisting of the following causes of death: 26 cases of isolated torso trauma, 22 cases of cerebral hypoxia and 22 cases of sudden cardiac death. Serum and CSF levels of BDNF, GFAP, NGAL, IL-6, S100B and NSE were obtained using quantitative immunoassays (Randox Laboratories, Crumlin, United Kingdom and ECLIA; Roche Diagnostics, Mannheim, Germany), a turbidimetric assay for ferritin (Tina-quant Ferritin Gen.4; Roche Diagnostics), and a kinetic absorbance test for LDH (Roche Diagnostics). Ethical approval was received by the Ethics Committee of the University of Leipzig (local code: 117-12-23012012). Figure 1 summarizes the findings of the individual markers from previous studies.

### 2.2. Statistical Analyses

Samples taken from CSF were included in the statistical analyses of the given study, as serum samples were only recommended for TBI detection in a minority of previous investigations [6,7,8,9]. Statistical analysis was performed using GraphPad Prism version 8 (GraphPad Software, San Diego, CA, USA), Microsoft Excel version 16.16 (Microsoft Corporation, Redmond, WA, USA) and the open-source software R (version 3.4.1, The R Foundation for Statistical Computing).

First, receiver operating characteristic (ROC) curves were created independently for CNS biomarkers and acute-phase proteins, including all 100 aforementioned cases, to identify sensitivity and specificity when investigating fatalities by single-marker measurements. Moreover, the ROCs were used to establish potential threshold values based on conservative estimations to discriminate between TBI cases and controls using bootstrap replicate confidence intervals. Positive likelihood ratios were calculated for each individual biomarker to assess their utility to screen for acute lethal TBIs. The accuracy was calculated as the sum of the true positive and true negative results, divided by the number of included cases. ROC curves were calculated to demonstrate individual diagnostic ability. However, using this maneuver to rank/rate the diagnostic ability between markers does not maximize the potential of each marker to work as a group.

Rather than comparing individual markers to find a single best marker, a classification tree model considers all the markers together and find the most effective way of differentiating/classifying TBI and controls. This would help to provide higher diagnostic accuracy than individual markers. Therefore, a classification tree model was performed based on all individual cases, where all eight biomarkers were analyzed using statistical learning analytical techniques performed with R. Tree models involve segmenting the predictor space into simple regions by building splitting rules which can be summarized in a decision tree. Single biomarkers/predictors would be a one-node classification tree model with the cut-point/threshold being the one obtained in the ROC. When considering multiple biomarkers/predictors, statistical learning methods were used to fit the optimal decision tree model. Initially, the recursive binary splitting method expanded the decision tree to be as large as possible. This would cause overfitting and an increase in variance. The expanded decision tree was subsequently pruned down using the cost complexity pruning method, which minimizes the turning parameter. The misclassification rate, an estimation of test error in statistic learning methods, was determined using K-fold (*k* = 5) cross-validation for the constructed tree models. This maneuver resulted in a total of 53 TBI and control cases (excluding the remaining samples of the two cohorts where few marker levels were randomly missing) and was done to assess the accuracy and misclassification rate of combined biomarker applications in TBI screening. It was determined whether there was any significant diagnostic improvement when combining different markers compared to the diagnostic accuracy of the analyses of individual markers.

Third, all biomarkers from the TBI group included in the decision tree models were correlated to screen for coherent changes of biomarkers related to acute lethal TBIs and Spearman’s rank correlation coefficient was reported.

## 3. Results

**Figure 2:**
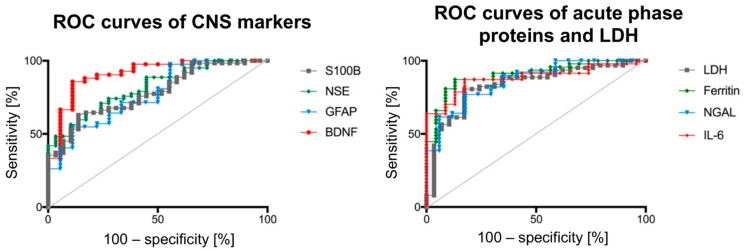
The receiver operating characteristic (ROC) curves for the biomarkers in this study are depicted. The survival times for TBIs and control cases were comparable (<2 h).

CSF biomarkers moderately to largely increase the likelihood to detect an acute lethal TBI. The 95% confidence intervals of the areas under the curve (AUC) of the ROC curves of all individual biomarkers were overlapping (Figure 2). The accuracies of the individual AUC curves were statistically comparable. For diagnostic purposes, BDNF, IL-6 and ferritin CSF levels provide the quickest options to distinguish TBI cases from controls when focusing on one individual biomarker. Table 1 summarizes the AUC and the 95% confidence intervals of the ROC curves for the investigated biomarkers, as well as the sensitivity: specificity ratio, accuracy and the positive likelihood ratio for a given threshold value following a threshold value analysis accentuated for specificity.

**Figure 3:**
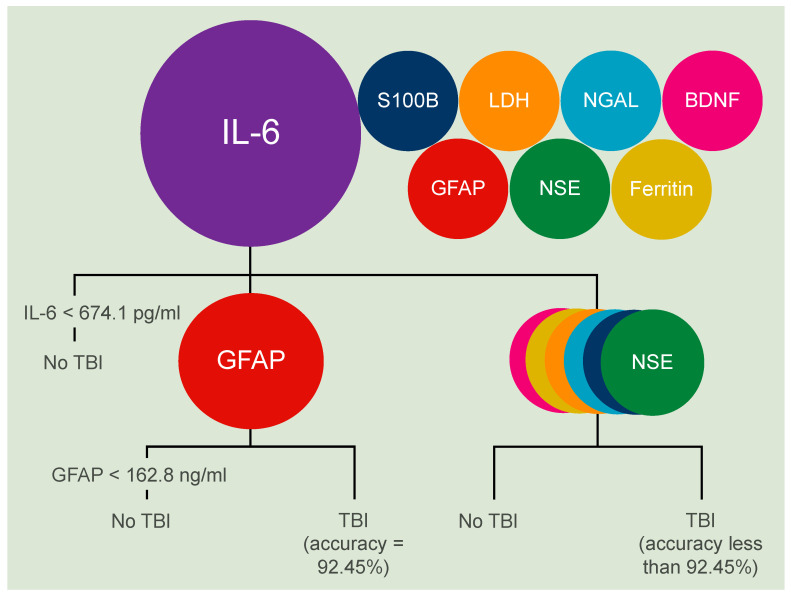
A graphical summary of the results of this study based on the decision tree modelling approach. From all the potential biomarker combinations in this study, the combination of IL-6 and GFAP resulted in the highest accuracy of 92.45% for the detection of an acute lethal TBI.

Combining IL-6 and GFAP helps correctly classifying lethal acute TBIs in more than 90% of cases. A combination of IL-6 and GFAP correctly classified 49 out of the given 53 cases, resulting in an accuracy of 92.45%, and a complimentary rate of misclassification of 7.55% by deploying the decision tree modelling approach (Figure 3). To achieve this high level of accuracy, the modelling approach gave a threshold value of 674.1 pg/mL for IL-6, which was higher compared to that for the individual marker (Table 1). The threshold value for GFAP of 162.8 ng/mL was comparable to that obtained for the individual marker (Table 1). The established nodes (breakpoints) were statistically significant. Adding a third marker to this combination or combining any further parameters to IL-6 resulted in no improvement to the given diagnostic accuracy.

### BDNF Significantly Correlates with Three Acute Phase Proteins in Acute Lethal TBIs

**Figure 4:**
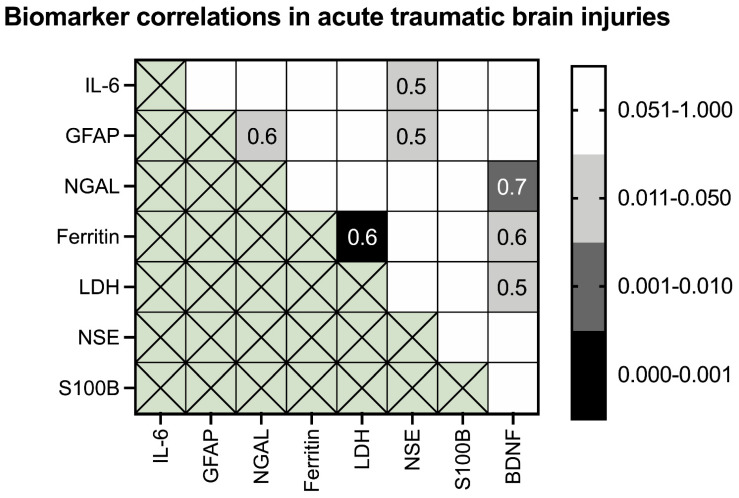
A heat map displaying the correlations between the cerebrospinal fluid biomarkers for the detection of acute lethal traumatic brain injuries. Significant *p*-values (*p* ≤ 0.05) are shown with different intensities of grey and black. For significant correlations, the correlation coefficient was added numerically within the respective boxes.

**Figure 5:**
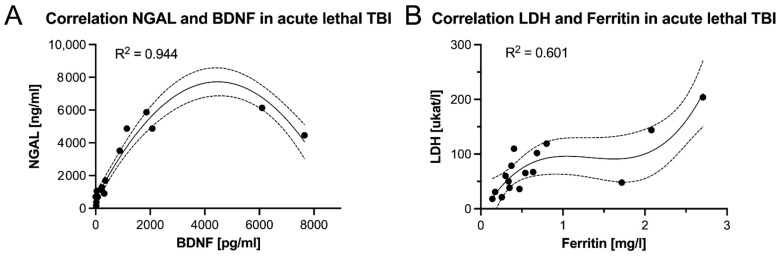
A polynomial regression analysis revealed (**A**) a strong correlation between neutrophil gelatinase-associated lipocalin (NGAL) and brain-derived neurotrophic factor (BDNF), and (**B**) a moderate correlation between lactate dehydrogenase (LDH) and ferritin in acute lethal traumatic brain injuries (TBI) using post mortem cerebrospinal fluid biochemistry.

Significant positive correlations were found between BDNF and the acute phase proteins ferritin (*p* = 0.049), LDH (*p* = 0.024) and NGAL (*p* = 0.002) (Figure 4). The latter correlation reached an R^2^ of 0.944 in a polynomial regression (Figure 5A) according to the following formula:

NGAL = −0.0004 × BDNF^2^ + 3.2118 × BDNF + 557.26

GFAP significantly correlated with NGAL (*p* = 0.021) and NSE (*p* = 0.033) (Figure 4). Moreover, NSE significantly correlated with IL-6 (*p* = 0.049) (Figure 4). A moderate correlation was also found for ferritin and LDH (*p* < 0.001) (Figure 4) with an R^2^ of 0.601 in a polynomial regression (Figure 5B) according to the following formula:

LDH = 7 × 10^−8^ × Ferritin^2^ + 0.0034 × Ferritin + 41.829

S100B was the only biomarker investigated to present totally independent changes from other co-analyzed biomarkers in acute lethal TBIs.

## 4. Discussion

The great value of continuing research related to TBI beyond the sole determination of death is exemplified by the chronic neurodegenerative disorder named chronic traumatic encephalopathy. The underlying disease linked to repetitive TBI remains obscure for clinicians, and can therefore only be diagnosed post mortem [3]. Vice versa, from the treatment-focused clinical point of view, it seems pointless both economically and sociologically to continue monitoring a patient or collecting laboratory results after death. However, the post mortem investigation of TBI-related biomarkers in stable body fluids such as CSF [22] might set useful reference points for more accurate outcome and mortality estimations in the medicolegal field in the near future. This given study focused on acute TBI-related deaths with a predefined maximum survival time of 2 h to establish biomarker values covering (i) the initial response of the organism to the traumatic impact as forensically important signs of vitality, and, (ii) a period in which the patient has not survived long enough for sufficient clinical diagnostics and therapy but allowing for initial emergency interventions such as the collection of body fluids for diagnostic purposes even in the pre-hospital setting.

For the cause of death determinations, forensic biomarkers to detect lethal acute TBIs could be of great advantage in cases for which external impact signs are missing. The assessment of eight TBI-related biomarkers performed here enables forensic pathologists to diagnose an acute lethal TBI with an accuracy of 92.45%, using CSF levels of IL-6 and GFAP with the cut off values stated in this study. Interestingly, both markers recently also showed promising results in the microscopic investigation of their neuronal and astroglial expression in fatally injured human brain tissue compared to controls [23]. When the two biomarkers are examined individually, taking the sensitivity and specificity values stated in this given study, GFAP and IL-6 accurately classify 78% and 86% of cases, respectively. For comparative reasons, using the recently established CSF cut off value of 385.5 ng/mL for GFAP which is independent of the TBI survival time [8], and was calculated without accentuation for specificity, the classification is accurate in only 70% of cases. This comparison indicates that individual cut off values will have to be established depending on the respective specific survival time and in consideration of statistical validity. Also, the results of the given study indicate that the cut off values have to be adjusted when used in combination to outperform individual markers with regards to accuracy.

The physiological level of IL-6 in antemortem CSF was stated to be 1 to 23 pg/mL, which was established based on 121 patients with no present or previous inflammatory disease [24]. However, a reference value for non-TBI cadaveric IL-6 levels in CSF is not available to date. In the rat model, an immediate increase of IL-6 in CSF following axonal injury was observed, with a peak occurring between 2 and 4 h after the event [25]. As lethal TBIs frequently also involve extracranial injuries and IL-6 is not CNS-specific [26], it could be argued that the IL-6 increase following lethal acute TBI, as in the presented study, is influenced by its increased release outside the brain as a systemic response to the traumatic damage. However, in a murine model, it has been shown that although IL-6 can cross the blood-brain-barrier into the CNS, it is transported via a saturable mechanism and was intact in only 17% of cases at most [27]. Thus, the vast majority of IL-6 detected in CSF samples of acute TBIs likely originates from the CNS itself, especially from cerebral astrocytes [28], neurons [23] or even microglia [29].

This study revealed that combining the acute phase protein IL-6 with the CNS marker GFAP increases the accuracy of detecting a lethal acute TBI and, therefore, our above-stated hypothesis can be accepted. Apart from its occasional presence in neurons [18,30] and testicular Leydig cells [31], the intermediate filament GFAP is almost exclusively expressed in astrocytes [32]. Thus, GFAP is specific for brain injury and may predict cerebral lesions even in polytrauma patients using biochemical methods [33]. Since IL-6 and GFAP can be detected in both serum [33] and CSF [8] samples immediately following an injury [34] or post mortem, they seem to be a suitable biomarker combination for the detection of acute lethal TBIs. This observation was further substantiated in this study. The concept of a combination of biomarkers to detect TBIs has so far been unexplored in the forensic field. Clinical investigations showed that the combined investigation of ubiquitin carboxy-terminal hydrolase-L1 and GFAP concentrations in the serum of 206 patients to detect TBIs resulted in an increased accuracy (here: AUC 0.94) compared the individual use of these markers [15]. However, a larger clinical investigation on 584 TBI patients revealed that a combined investigation of ubiquitin carboxy-terminal hydrolase-L1 (UCH-L1) and GFAP could not outperform GFAP on a statistically significant level [34]. Moreover, the combination of serum S100B and apolipoprotein A1 maximized the classification accuracy for mild TBIs compared to the individual biomarkers in a clinical setting [35]. Until now, ubiquitin carboxy-terminal hydrolase-L1 has not been assessed post mortem for this purpose, to the best of the authors’ knowledge.

According to the positive likelihood ratio presented in this study, GFAP moderately increases the probability of the presence of an acute lethal TBI by 30% [36], if the CSF concentration reaches 134.4 ng/mL. The remaining biomarkers further increase the probability of a lethal acute TBI by more than 45% [36] given their high positive likelihood ratios. For comparative reasons only, positive likelihood ratios for widely used clinically established cancer screening tests are far less than those for the TBI biomarkers in the given study, except for GFAP. Serum prostate-specific antigen to detect prostate carcinomas has a positive likelihood ratio of 1.85 [37], the Guaiac test used for bowel cancer screening has a positive likelihood ratio of 2.26 [38], and the screening mammography for the early detection of breast cancer has likelihood ratios ranging between 5.2 and 8.8 depending on the screened age group [39]. Consequently, applying the here used biomarkers to screen for acute lethal TBIs in a forensic setting seems appropriate, especially once the proposed combination of the acute phase protein IL-6 and the CNS marker GFAP is used.

The given study highlights the potential to increase the diagnostic accuracy of a medical condition based on biomarker combinations in body fluids that are routinely sampled and stored during forensic autopsies [8]. Although not routinely done in our departments, CSF sampling prior to or instead of an autopsy could be done with minimal effort. The practical use of such methods is predominantly hampered by particular regional laws rather than technical feasibility. Possibly, the diagnostic accuracy of 92.45% in the given example could be further increased by adding additional biomarkers to the decision tree beyond the eight investigated here, up to a point where virtual certainty is reached. Beyond the here investigated biomarkers, there are few that were described as being diagnostic in the clinic but have not yet been investigated forensically, e.g., the aforementioned ubiquitin carboxy-terminal hydrolase-L1 or αII-spectrin breakdown products [3].

On a greater scale, being able to assess a multitude of diseases from body fluids in combination with advanced computer evaluations of virtual autopsies [40] could decrease the workload of forensic pathologists in the future, while at the same time enabling them to focus on more complex, time-consuming cases to gather valuable evidence for later trials. This is of special interest in areas where autopsy caseloads exceed the available forensically qualified personnel [41] or as a screening tool in countries where autopsy rates are low for religious, ethical or socioeconomic reasons. It has been suggested previously that establishing large biochemical databases [16] as a side-product of routine autopsies could enhance pathophysiological knowledge and ultimately create a new field of “biochemical evidence”. Costs that arise from the creation of such databases could be limited by gathering additional knowledge on the relationship between several biomarkers, enabling the prediction of a particular biomarker level from data on others in the related disease. In the given study, BDNF and NGAL levels in cases of acute lethal TBIs were highly correlated, and the respective counterpart could be deduced from applying the formula stated in this study.

It should be noted that the routine applicability of post-mortem biochemical observations will likely be limited to the early phase after death due to tissue degradation or the stability of the biomarker in the respective body fluid [16]. The increased levels of both IL-6 [7] and GFAP [8] within up to 150 h after death indicate reasonable stability of the favored markers to detect acute lethal TBIs in CSF. Typically, cadaveric blood is more prone to autolytic and proteolytic changes compared to CSF and some serum markers are unstable even within 48 h after death [42,43]. However, both GFAP and IL-6 remain evenly stable post mortem serum parameters for the given post mortem delay [42]. It is likely that here stated post mortem CSF concentrations of GFAP and IL-6 exceed the clinical levels of these biomarkers in severe TBIs as the lethal outcome might initially be associated with a greater amount of brain damage and at the time of death will most likely be “boosted” by general cell death in fatal outcomes [6].

In collaboration with biochemistry units, post mortem biochemistry could be established as an additional diagnostic tool for external autopsies, which are performed away from the forensic departments by independent investigators, and in the long-term increase diagnostic accuracy. The majority of biomarkers can be measured with minimal effort in a time-efficient and cost-effective way using automated analyzers. Biomarkers appear to be economically superior compared to other methods, such as forensic imaging. Additionally, future studies should explore the strategy to combine forensic biochemistry with minimal invasive autopsies [44] to further refine and establish this diagnostic option.

### 4.1. Limitations

First, the study is limited by its sample size. Second, unswayable factors such as the ambient temperature at the time of death and the freezing of the used body fluids for storage purposes might have impacted degradation processes and, thus, the biochemical observations in the given study. However, these limitations would also apply in further practical uses, i.e., the markers presented here seem robust enough to allow for clinical application. Third, undetected neurodegenerative conditions might have impacted the concentrations of the here investigated TBI-related biomarkers [26], although the case selection was done following strict criteria with the exclusion of chronic CNS diseases in all involved studies [6,7,8,9,22,23,42,43]. The authors believe that the chosen controls, which contained causes of death that deprived the oxygen supply to the brain such as hanging or strangulation, are representative of a forensic setting. However, other brain-affecting causes of death such as strokes or brain tumors were not considered and should, therefore, be part of future studies. The biomarker selection for this post mortem study was initially made based on promising results to discriminate TBI cases from controls in a clinical setting. Limited funds during the course of this study prevented testing additional promising biomarker combinations including UCH-L1, microtubule-associated protein tau or neurofilament L.

### 4.2. Conclusions

This study provides evidence that acute lethal TBIs are detectable with high statistical accuracy (up to 92.45%) using forensic biochemistry. The high inter-individual correlations of biomarkers may help to estimate the CSF concentration of an unknown biomarker based on other markers. CNS biomarkers can be combined and interpreted together, and this may increase the diagnostic accuracy of acute lethal TBI. The methods applied here can also be used to stratify and prioritize some methods/samples over others due to increased reliability, which is applicable beyond the forensic investigation of traumatic fatalities in the future.

## Figures and Tables

**Table 1:** A threshold value analysis (overall diagnostic accuracy, accentuated for specificity) for the biomarkers shown in Figure 2 is depicted. The determined threshold values are depicted in blue. AUC = area under the curve; TBI = traumatic brain injury.

Biomarker	AUC	95% Confidence Interval	Threshold Value	Sensitivity: Specificity Ratio [%]	Positive Likelihood Ratio	Accuracy [%]
TBI biomarker of the central nervous system						
S100B	0.78	0.69–0.88	2267 ng/mL	37.1:96.6	10.76	79
NSE	0.82	0.73–0.90	598.5 ng/mL	48.4:96.5	14.03	83
GFAP	0.77	0.64–0.89	134.4 ng/mL	40.5:94.4	7.29	78
BDNF	0.91	0.83–0.99	11.1 pg/mL	64.3:95.7	14.68	86
Acute phase proteins as TBI biomarkers						
IL-6	0.88	0.80–0.96	99.1 pg/mL	63.8:95.7	14.68	86
Ferritin	0.91	0.83–0.98	1.73 mg/L	66.0:95.7	15.17	87
LDH	0.84	0.75–0.93	16.71 µkat/L	41.9:96.5	12.16	81
NGAL	0.86	0.76–0.96	334.4 ng/mL	61.5:94.1	10.46	84
